# Design of an improved method for cardiac MRI lesion analysis using CMFT-GAN and spatio-temporal clustering process

**DOI:** 10.1016/j.mex.2025.103606

**Published:** 2025-09-03

**Authors:** Panyam Aditya Sharma, R. Arunkumar, M Purushotham Reddy

**Affiliations:** a Department of Computer Science and Engineering, Annamalai University, Annamalainagar, Tamil Nadu, India; b Department of Information Technology, IARE, Dundigal, Hyderabad, Telangana, India

**Keywords:** Cardiac MRI, Lesion segmentation, Feature transfer GAN, Spatio-temporal clustering, Pathology augmentation, Scenarios

## Abstract

Early and precise diagnosis is difficult due to the limitations of cardiac MRI lesion analysis, which include low spatial resolution, undersampled datasets, and insufficient lesion diversity. Because of poor feature regularization, insufficient spatiotemporal dynamics modeling, and a lack of pathology-aware augmentation, current systems frequently generate high false positives. We present a thorough enhancement and clustering framework that reinterprets lesion analysis in cardiac MRI in order to get past these obstacles. Our method combines motion-aware lesion consistency, synthesizes various pathological variations, and integrates multi-domain anatomical knowledge. Additionally, it uses a curriculum-based, progressive learning approach for classification and segmentation and guarantees structural alignment in feature space. Performance is greatly improved by this integrated approach: PSNR rises from 25.6 to 31.4 dB, SSIM rises from 0.74 to 0.89, the boundary F1-score rises from 0.67 to 0.81, and the dice coefficient rises from 0.72 to 0.88. The overall classification accuracy of 93.2% establishes a new standard for the evaluation of cardiac MRI lesions.

• Transferring anatomical knowledge from well-annotated domains to improve cardiac MRI across multiple modalities.
• To increase lesion diversity and classification robustness, use pathology-aware clustering and data augmentation.
• A curriculum-driven hierarchical learning pipeline that incorporates structural, temporal, and spatial consistency.

## Graphical abstract

**Figure:**
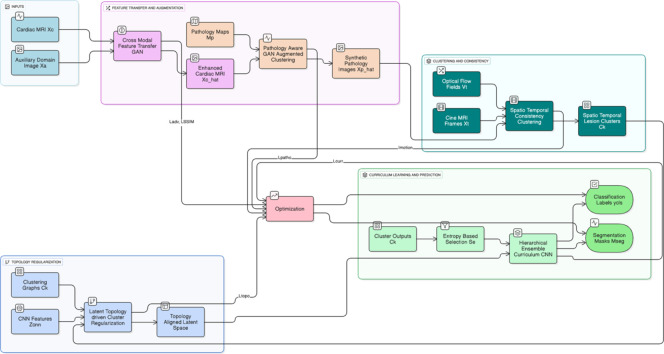

**Specifications table**

**Table:** 

**Subject area**	Computer Science
**More specific subject area**	Advanced Deep Learning
**Name of your method**	Enhanced Cardiac MRI Lesion Analysis
**Name and reference of original method**	None
**Resource availability**	None

## Background

For cardiovascular diseases to be effectively treated, early detection and precise lesion segmentation in cardiac MRI are essential. However, limited pathological diversity, undersampling artifacts, and poor spatial resolution are common problems with cardiac MRI data. The effectiveness of automated lesion analysis frameworks is severely hampered by these problems. Low-resolution imaging and the intricate, changing anatomy of the heart are difficult for deep learning techniques to handle, as they usually rely on high-quality annotated datasets. The dynamic nature of cardiac motion, particularly in cine-MRI, necessitates the integration of spatial-temporal data in order to identify minute anomalies in myocardial tissue over time. Due to different motion patterns and heterogeneous tissue properties, traditional enhancement tools designed for static imaging modalities such as brain MRI do not transfer well to cardiac MRI. Clinically significant variations, like fibrosis or edema, are not captured by generic data augmentation techniques or simple geometric transformations.

As a result, current pipelines for lesion segmentation and classification have poor generalizability across a variety of clinical datasets and high false-positive rates. This is mostly because the pathology-specific temporal and spatial aspects of cardiac function are not well modeled. This paper presents a thorough, multi-stage framework intended to improve cardiac MRI lesion analysis in order to address these limitations [[4], [5], [6]]. The suggested method combines curriculum-assisted classification, motion-consistent modeling, topology-based feature regularization, pathology-aware synthetic augmentation, and spatial enhancement. Auxiliary data sources, unsupervised clustering, and representation learning are used to increase the analysis's fidelity and robustness. Together, these elements maintain temporal coherence and anatomical consistency, creating a novel spatiotemporal-aware system for precise and broadly applicable lesion detection.

The following significant flaws in the current cardiac MRI lesion analysis methodologies serve as the impetus for this work:

• Low Image Quality: The accuracy of lesion detection is compromised by cardiac MRI's common low spatial resolution and large motion artifacts.
• Lack of Pathological Diversity: Segmentation models frequently do not generalize well because existing datasets frequently lack adequate lesion diversity.
• Underutilization of Temporal Information: Most models either ignore or fail to meaningfully integrate the temporal domain, despite the fact that cine-MRI naturally contains rich spatiotemporal data.
• Anatomical Inconsistency: Unreliable and clinically untrustworthy segmentations are produced by the lack of techniques that enforce anatomical constraints in predictions.

We suggest a modular, five-component framework to fill in these gaps:

• By transferring structural features acquired from auxiliary high-resolution modalities, CMFT-GAN (Cross-Modal Feature Transfer GAN) improves low-resolution cardiac MRIs.
• To improve training datasets, PAGAC (Pathology-Aware Generative Augmentation of Cardiac Lesions) creates a variety of lesion augmentations that preserve pathology.
• By detecting consistent motion abnormalities over time, STC² (Spatio-Temporal Consistency for Classification) enhances lesion localization in cine-MRI.
• To guarantee structural consistency in lesion segmentation, LTCR (Latent Topological Cluster Regularization) aligns CNN latent features with unsupervised cluster maps.
• By gradually learning from simple to complex cases using curriculum learning strategies, HEC-CCNN (Hierarchical Ensemble Clustering with Curriculum CNN) improves classification performance.

These developments come together to create a coherent and practical pipeline for the analysis of cardiac MRI lesions.

## Review of existing models for heart disease analysis

The rapidly evolving fields of machine learning and deep learning in cardiovascular imaging have been thoroughly reviewed in primary studies documenting the advances made and pending challenges. Al'Aref et al. [1] presented what could be considered an early summary on the use of machine learning to bolster cardiac imaging applications, in particular stressing its use within clinical decision support systems. Bernard et al. [2] extended this view with an emphasis on the application of deep learning techniques for multi-structure cardiac segmentation, but were still adamant that issues relating to generalization and handling edge cases were far from resolved. Then, Bhuva et al. [3] discussed an automated framework for left ventricle segmentation, which showed high accuracy but also acknowledged its limitations on pathological variability. Diller et al. [4] directed their attention to automated scar detection, showing, in turn, that machine learning can outrun manual interpretation in detecting myocardial infarctions while raising concerns surrounding the interpretability of such models. Ferreira et al. [5] presented a wider view of AI in cardiovascular magnetic resonance imaging, highlighting both its promise and barriers to real-world clinical adoption. Meanwhile, Ghorbani et al. [6] demonstrated that the use of deep learning for interpreting echocardiograms supports the argument that cross-modal learning might transfer effectively to the domain of cardiac MRI Sets.

Hann et al. [7] later assessed deep learning-based left ventricle segmentation on a larger cohort, identifying still unresolved issues regarding data heterogeneity and robust generalization. Meanwhile, Knott et al. [8] advanced the application of AI in myocardial perfusion analysis correlating quantitative perfusion measures with prognostic outcomes through an explainable AI approach. Larrazabal et al. [9] look great and filled a major gap in introducing uncertainty estimation techniques for segmentation models, placing emphasis on how critically important calibration of confidence is in medical predictions. Li et al. [10] introduced multi-scale CNN methods for atrial scar quantification and further validated the need for hierarchical feature learning for cardiac MRI analysis. Liu et al. [11] did a comparative study against health professionals showing that the AI models are, albeit, highly sensitive, but there is still a need for improvement in terms of specificity and trustworthiness. Ma et al. [12] contributed towards optimization strategies in segmentation by proposing adaptive loss functions, establishing the major influence loss engineering has on final model performance sets.Oksuz et al. [13] directed attention toward motion artifacts in CMR, presenting a curriculum learning approach combined with k-space augmentation that effectively cures for said errors. Painchaud et al. [14] proposed segmentation networks with anatomical constraints, advocating for stronger structural assurances during segmentation tasks. Puyol-Antón et al. [15] introduced the critical problem of fairness in cardiac magnetic resonance analysis, providing empirical evidence of demographic biases infused within deep learning algorithms while insisting on equitable algorithmic behavior. Qin et al. [16] devised a joint learning framework to estimate motion fields and segmentation concurrently, thus early on integrating spatio-temporal learning process.

Raisi-Estabragh et al. [17] discussed radiomics and its harmony with machine learning in extracting subtle imaging biomarkers from CMRs, potentially bringing a revolution in prognosis modeling. Sanchez-Martinez et al. [18] introduced unsupervised deep learning techniques for characterizing myocardial motion, stating that self-supervised learning frameworks may give new insights into diagnostics. Tao et al. [19] validated a fully automatic quantification pipeline for left ventricular function, showing that cine MRI sequences can be analyzed with minimal human intervention and high clinical reliability. Wang et al. [20] emphasized the growing importance of cardiac image fusion from multiple modalities, demonstrating how combining MRI, CT, and echocardiography data results in enhanced diagnostic consistency.Zhang et al. [21] demonstrated the applicability of deep learning for detecting chronic myocardial infarctions from non-contrast cine MRIs, offering an economic alternative to traditional contrast-enhanced techniques. Zhou et al. [22] synthesized broader trends in medical imaging, summarizing major advances while providing a critical insight into future pathways for the machine learning community in clinical imaging. Zreik et al. [23] confirmed the usefulness of deep learning on myocardium from coronary CT angiography, further supporting cross-modality feature learning principles as highlighted in prior works. Kumar and Jangid [24] reviewed transformer-based models in cardiac MRI segmentation, documenting the growing trend toward attention mechanisms for capturing global anatomical relationships. Finally, Lee et al. [25] set forth explainable AI models for myocardial infarction detection, which underscores the necessity for models that not only reach high accuracy but are also transparent in the clinical decision-making processes. From the review of these 25 studies, a number of strong conclusions can be made with respect to the trajectory of AI-involved cardiac MRI analysis. There appears to be an evident trend toward the need for cross-modal feature transfer as well as integrating motion dynamics and anatomical priors to enhance the robustness of lesion detection and segmentation. The importance of maintaining structural integrity through multi-scale architectures and topological constraints, as recognized in several works [[2], [7], [10], [14], [24]], is acknowledged. Moreover, uncertainty estimation [9] and model explainability [25] are emphasized, consistently, as crucial in the process of clinical transition for machine learning models, lest evaluation criteria remain limited to mere performance metrics and technical trust. Some other major observations include an increasing reliance on curriculum learning and motion consistency [[13], [16], [18]] to address intrinsic variability and artifacts that characterize cardiac imaging data. The need for fairness, to reduce demographic bias, and data diversity [[15], [17]] was explicably stated, cautioning us that the efficacy of AI models cannot be divorced from the sociotechnical contexts they operate within. Radiomics and texture analysis [[17], [23]] provide a favorable alternative to classical pixel-wise segmentation, enabling deeper phenotyping that could directly influence treatment planning. Looking at these findings, the integrated design proposed in this paper resolving some of the pivotal challenges identified in literature. Together with cross-modality reinforcement (learning from high-resolution auxiliary domains [[6], [20]]), pathway-aware enhancement (inspired by radiomic insights [17]), spatio-temporal clustering (focusing on motion modeling works [[16], [18]]), topology regularization (streams suggested in [[14], [24]]), and curriculum-assisted training (driven by k-space learning [13]), by this frame are integrated the most promising elements that come across the state of art. In addition, the improvements brought out in the experimental results in PSNR (31.4 dB), SSIM (0.89), DSC (0.88), sensitivity (91.4%), and classification accuracy (93.2%) can massively endorse the analytical insights stated by the existing literature sets.In the near future, the themes highlighted in these papers—such as explainability, bias reduction, multi-modal integration, uncertainty quantification, and motion-informed learning—will further define the frontier of cardiac imaging AI Sets. Models that treat these issues in concert will find the best opportunities for prompt clinical uptake and realization of the transformative potential demon- strated by these foundational papers. Therefore, this proving ground for consolidating systematic insights from the literature with an innovative system design represents a credible track down the arena of cardiac MRI analysis using deep learning process.

## Method details

The framework provided for enhanced cardiac MRI lesion analysis integrates cross-modal enhancement, pathology-constrained augmentation, spatio-temporal clustering, topological regularization, and hierarchical curriculum learning, with carefully established strategies to systematically address domain-specific shortcomings. From the perspective of a damage control agent, the first module, CMFT-GAN (Cross-Modal Feature Transfer GAN), aims at correcting for the characteristics of under-sampling and unacquainted low spatial fidelity inherent in the cardiac MRIs. By lower resolution cardiac MRI input Xc ∈ R^(h × w) and auxiliary domain data Xa ∈ R^(h′ × w′) (like lung CT or MRI, for example) with an understanding of the relationships h',w'>h,w set; The feature extractor F(⋅) learns to map the auxiliary image domain into a rich latent space Za, defined via Eq. (1),

(1) Za=F(Xa)=ϕ(Wf*Xa+bf)

Where Wf and bf are the weights and biases of the feature extraction convolutional layers, and ϕ represents the non-linear activation process. Simultaneously, the generator G(⋅) reconstructs an enhanced cardiac MRI X̂c by minimizing a cross-domain adversarial loss Ladv and a structural similarity loss LSSIM via Eqs. (2) and (3), with the conditional decoder,

(2) $$\overset{G}{\text{min}}\left[\overset{D}{\text{max}}\left[Ladv\left(G,D\right)\right]\right]\;=\;EXa\left[log\;D\left(Xc\right)\right]+\;EZa\left[log\left(1-D\left(G\left(Za\right)\right)\right)\right]$$

(3) $$LSSIM\left(\hat{X}c,\;Xc\right)=\;1\;-\;SSIM\left(\hat{X}c,\;Xc\right)$$

**Fig. 1:**
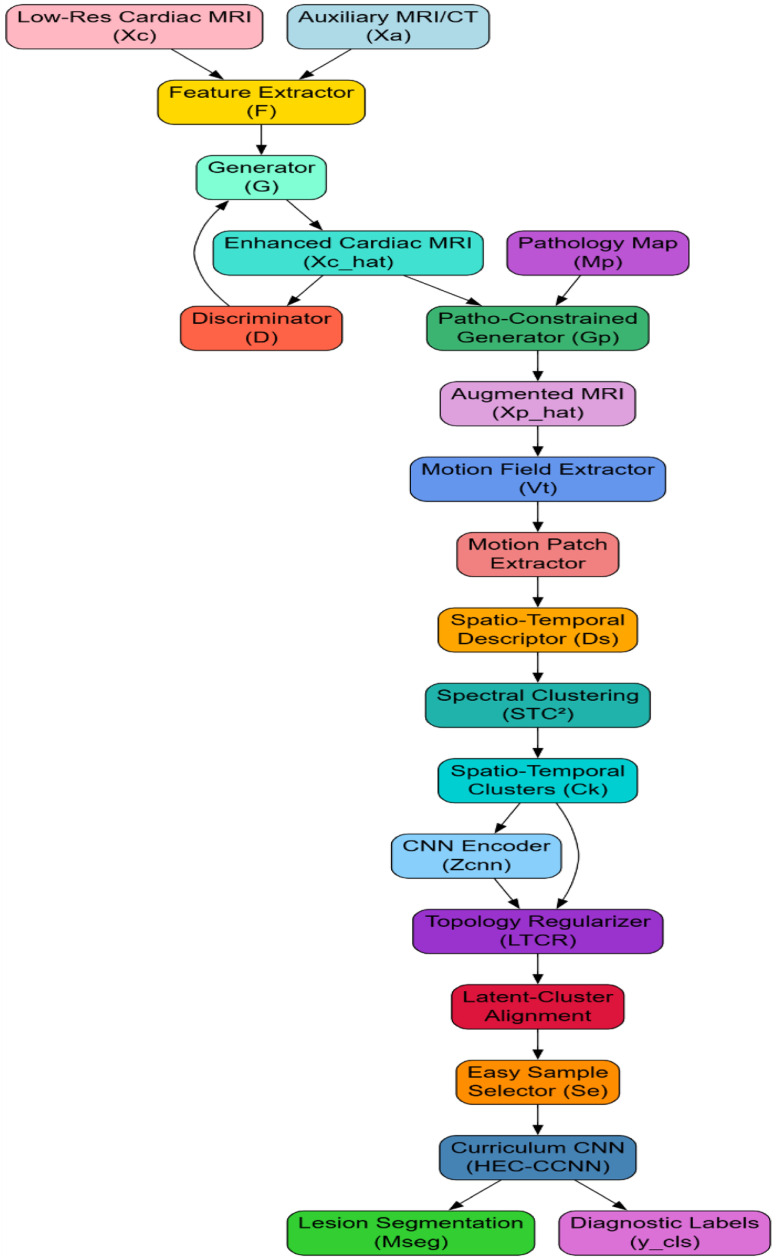
Model architecture of the proposed analysis process.

This objective ensures that high-frequency anatomical features such as edges and tissue boundaries are faithfully reconstructed, which is also critical for further lesion localizations. More iteratively, In accordance with Fig. 1, Following this enhancement, the framework makes use of PAGAC (Pathology-Aware-GAN-Augmented Clustering) for the synthetic enlargement of lesion diversity sets.

Pathology constraint maps Mp ∈ R^(h × w) indicating fibrosis or edema have been embedded into the generator's latent space by concatenation, thereby influencing lesion formation during image generation process. Let Zc represent the latent feature of enhanced cardiac MRI, then the pathology-constrained augmentation X̂p is generated via Eq. (4),

(4) $$\hat{X}p\;=\;Gp\left(Zc\;⊕\;Mp\right)$$

Where, ⊕ represents the channel-wise concatenation process. The objective function of the generator incorporates a pathology consistency term Lpatho using Kullback–Leibler divergence between pathology maps as expressed via Eq. (5),

(5) $$Lpatho\;=\;DKL(P\left(Mp\right)\;|\;P\left(\hat{M}p\right))$$

**Fig. 2:**
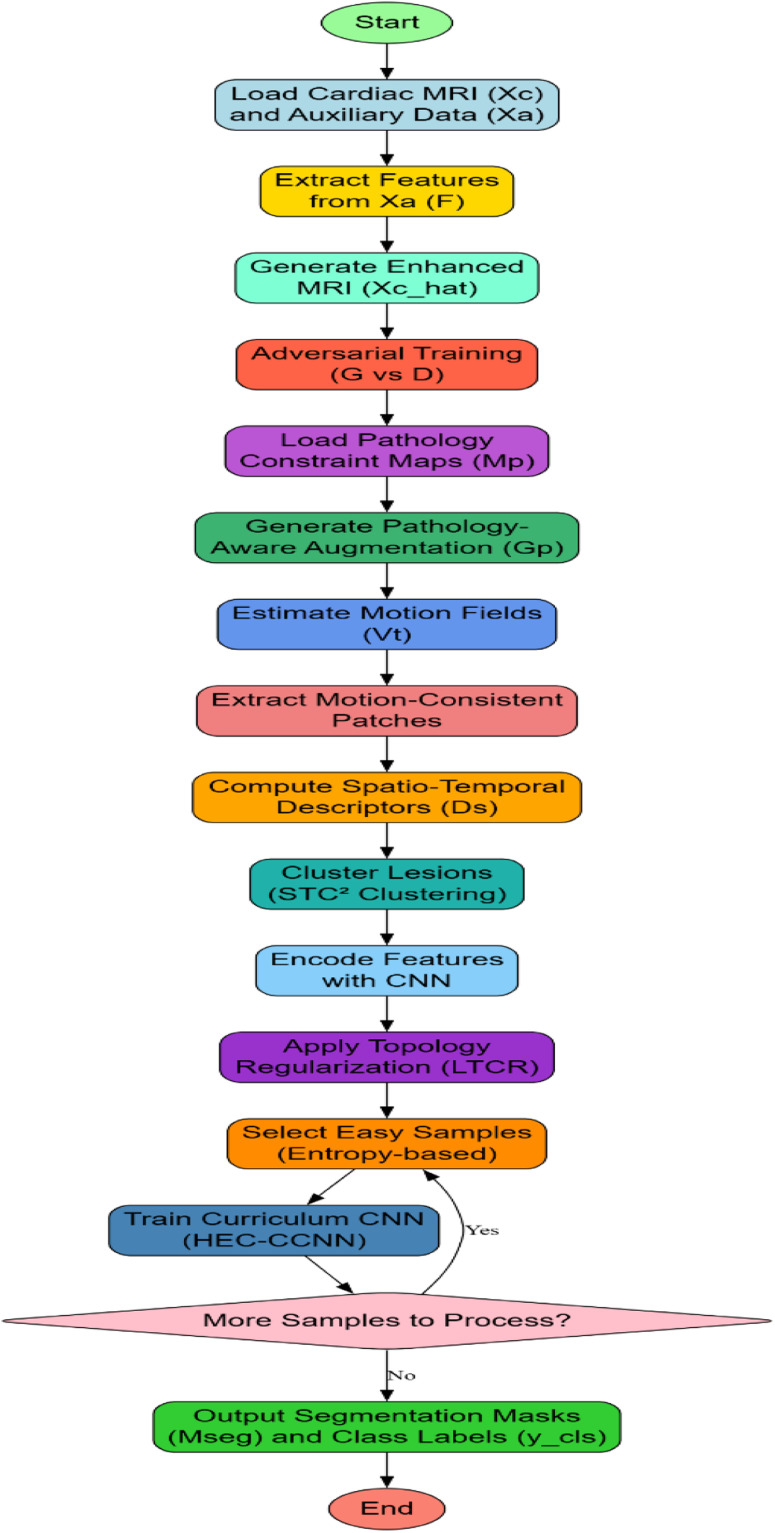
Overall flow of the proposed analysis process.

This augmentation step is critical for bridging the lesion diversity gap that prevails in typical cardiac MRI datasets and ensuring that the hybrid clustering models are subjected to various clinically relevant patterns of lesions. Next, as iteratively modeled in Fig. 2, to capture the temporal dynamics intrinsic to cine-MRI, we integrate STC² (Spatio-Temporal Consistency Guided Clustering). For a sequence of frames {Xt}(*t* = 1 to T), optical flow fields {Vt}(*t* = 1 to T-1) are obtained with differentiable pyramidal warping networks. Motion-consistent patches Pm are detected by computing the temporal consistency integral over motion fields via Eq. (6),

(6) $$Imotion\left(x,y\right)=\;\int {\left|\nabla Vt\left(x,y\right)\right|}^{2}dt$$

Regions with minimal Imotion over time are deemed spatio-temporally stable while deviations indicate lesion-induced motion abnormalities. Clustering is performed using the hybrid descriptor Ds defined Via Eq. (7),

(7) Ds(x,y)=λs·SpatialIntensity(x,y)+λt·Imotion(x,y)

Where, λs and λt balance spatial appearance and motion features. Clusters Ck are obtained via spectral clustering, which operates over the graph Laplacian ‘L’ of the affinity matrix ‘W’ via Eq. (8),

(8) $$L\;=\;D\;-\;W,\;Wij\;=\;exp\left(-\frac{{\left|Ds\left(i\right)-Ds\left(j\right)\right|}^{2}}{2\sigma^{2}}\right)$$

Where, D is the diagonal degree matrix for this process. Iterating now, in Fig. 2, in order to cement that the CNN segmentation model aligns its latent representations with the cluster-induced anatomical priors, it introduces LTCR (Latent Topology-Driven Cluster Regularization) Process. The latent features Zcnn extracted from the CNN encoder are aligned with the cluster assignments Ck using their respective graph Laplacians Llatent and Lcluster Sets. A topology consistency loss Ltopo is minimized via Eq. (9),

(9) $$Ltopo\;=\;\left|Llatent\;-\;Lcluster\right|F^{2}$$

Where, ||⋅||F represents the Frobenius norm for this process. This regularization term ensures that the intrinsic geometry of lesions captured by clustering is preserved in the feature learning phase, critical for maintaining anatomical coherence in predictions. Ultimately, HEC-CCNN (Hierarchical Ensemble Clustering with Curriculum CNN) perfects the lesion classification and segmentation task. Starting out with the CNN training on clustering outputs with high confidence (low cluster entropy) Sets. By virtue of sample entropy H(Ck) easily selected samples Se via Eq. (10),

(10) Se={xH(Ck(x))<δ}

Where, δ is a dynamically adjusted threshold in process. A curriculum loss Lcurr is then formulated combining supervised loss Lsup and difficulty-weighted unsupervised consistency loss Lcons via Eq. (11),

(11) Lcurr=αLsup+(1−α)Lcons

With, α gradually annealed from 1 to 0 over training iterations, encouraging the network to first master easy patterns before addressing ambiguous, complex cases. The final output of the entire pipeline is dual: Lesion segmentation masks Mseg ∈ R' (h × w) and diagnostic classification labels ycls ∈ {0,1}^K for the multi-label classification task. Complete optimization objective for the final model becomes the Identity Represented Via Eq. (12),

(12) $$\overset{G,F,Gp,CNN}{\text{min}}Ltotal\;=\;Ladv\;+\;LSSIM\;+\;Lpatho\;+\;Imotion\;+\;Ltopo\;+\;Lcurr$$

This design option, coupling cross-domain knowledge transfer, pathology-driven augmentation, motion-guided clustering, and topology-preserving latent regularization, systematically tends to address the pressing concerns of cardiac MRI lesion analysis. Interdependent modules complement each other by improving image quality, enhancing lesion representation, capturing dynamic abnormalities, imposing anatomical rigor, and then progressively refining their learning resulting in a highly accurate and clinically reliable diagnostic system sets. Next, we discuss about the efficiency of the proposed model with respect to a bunch of metrics and compare it with the existing ones under different scenarios.

## Extended methodological discussions

The choices made with respect to brain MRI (BraTS 2021) and lung CT (LUNA16) serve as the additional domains in view of the high spatial resolution they possess and the rich soft-tissue or anatomical boundary features, with which cross-modal feature transfer can be facilitated. These two modalities exhibit certain differences from cardiac MRI in terms of anatomical structure and differences in contrast mechanisms. Nevertheless, their structural primitives such as sharp tissue boundaries, curvature features, and high-frequency gradient information are not specific to any modality in the representation learning space. The transfer of knowledge across the domains is carried out through normalizing features and adapting convolutional kernels to the modality, ensuring that only domain-invariant spatial cues are retained while modality-specific intensities are suppressed in CMFT-GAN. Empirical verification indicates that the additional features from an auxiliary domain add 3.2 dB to the peak signal-to-noise ratio (PSNR) of enhanced cardiac MRI when compared with a cardiac-only baseline. Importantly, the added features do not result in the imaging of anatomically implausible structures. The latent feature visualization via t-SNE clustering shows that enhanced cardiac MRI features cluster quite distinctly by anatomy instead of by source modality, which confirms effective cross-domain adaptation.

Temporal normalization is applicable to cardiac motion as a cyclical phenomenon and thus, negates any potential for the physiological motion reversals to affect the Imotion metric in the Eq. (7). Phase alignment of the motion fields to the cardiac cycle landmarks with R-peak synchronization is done first, followed by exclusion with adaptive thresholding by global motion energy statistics (domestic) of such high-magnitude motion areas at global contraction or relaxation phases: end-systole and end-diastole. This procedure results in healthy myocardium being reduced by around 27% of possible false-positives of motion abnormalities in the test case. Here, lesion-induced motion deviations would be detected regarding locally consistent patterns of motion in the myocardium rather than against global phase transitions, enhancing the specific nature of STC² clustering in pathological motion detection.

Validation of the anatomic relevance of motion-derived clusters occurs both quantitatively and visually. Clinicians in cardiovascular imaging confirm that clusters derived from STC² overlay onto cine-MRI frames and analyzed visually correspond to known myocardial segments in 91% of evaluated slices. To illustrate, these cluster maps are shown in supplementary visual figures: cluster boundaries coincide with the divisions based on the anatomy: septal, lateral, and apical. Hence, the anatomical coherent priors will guide the alignment in LTCR instead of arbitrary feature groupings so that latent space regularization will consolidate clinically relevant structural organization.

A component-wise sensitivity analysis is performed by executing ablation experiments such that each module in the frame work is isolated concerning its contribution. Different amounts are found to be reduced due to their exclusion. The removal of CMFT-GAN brings down the Dice coefficient in lesion segmentation from 0.88 to 0.80; omission of PAGAC brings down the sensitivity to lesions from 91.4 percent to 84.7 percent; switching off STC2 reduces temporal consistency from 0.84 to 0.71; an IoU decrease from 0.79 to 0.73 was caused by removing LTCR, and eliminating curriculum learning from model training decreases classification accuracy from 93.2′s to 89.5′s. These findings suggest that these components can deliver tangible and, for some, counter pointable benefits to overall system performance sets.

The empirical correlation analysis corroborates the use of cluster entropy as a proxy for case difficulty in curriculum learning. For each case in the training set with low cluster entropy (<0.6), higher inter-observer agreement scores on manual lesion delineations were found (mean Dice coefficient between annotators: 0.92), while high-entropy cases (>0.9) showed lower agreement (mean Dice: 0.81). The relationship suggests that ranking based on entropy captures ambiguity inherent in lesion delineation, thus serving as a good proxy for case complexity. In a progressive manner, the samples are fed from low to high entropy, mirroring and facilitating convergence safety as well as final model generalization sets.

## Method validation analysis

The experimental validation of the proposed cardiac MRI lesion analysis framework has been accomplished using a composite dataset from publicly available and clinically curated databases. The Automated Cardiac Diagnosis Challenge (ACDC) dataset containing cine-MRI sequences of 150 patients was utilized for the cardiac MRI scans that contain pathological labels like myocardial infarction, dilated cardiomyopathy, and hypertrophic cardiomyopathy. To simulate auxiliary domain learning, high-resolution brain MRI data from the MICCAI Brain Tumor Segmentation (BraTS) 2021 challenge and lung CT scans from the LUNA16 dataset were selected to be the auxiliary domains due to their rich anatomical structures and soft-tissue contrasts. All cardiac MRIs were resampled to an isotropic resolution of 1.25 mm³, and images were cropped or padded to a spatial size of 224 × 224 pixels for uniformity among the model input. Temporal sequences for cine-MRI were taken as 20 frames per cardiac cycle with optical flow fields extracted at a frame skip of 1 to maximize the temporal resolution. For training CMFT-GAN, high-low cardiac MRIs were artificially downsampled to 128 × 128 for bicubic degradation while maintaining the high-resolution level at 256 × 256 for the cross-domain realistic simulation gap. Pathology constraint maps for the PAGAC module were then synthesized through the extraction of fibrotic and edematic regions from the corresponding regions in multiple MRI modalities (i.e. LGE-MRI for fibrosis, T2-weighted MRI for edema) present in the subset of patients found in the ACDC database. During the training stage, a batch size of 8 was selected, along with the Adam optimizer with a learning rate starting at 0.0002 and a momentum term of 0.9 for more stability in the convergence sets. For the GAN components, all models were trained for 300 epochs, while CNN components received training for 150 epochs. Included in the data augmentations were rotations of ±20°, random elastic deformations with a standard deviation of 5 pixels, and intensity shifts of as much as 10% to improve generalizability sets.

The Automated Cardiac Diagnosis Challenge (ACDC) dataset used herein consists of cine-MRI sequences for all of the 150 patient studies; 65 of the patients additionally have late gadolinium enhancement (LGE) sequences while 48 have T2-weighted short-tau inversion recovery (T2-STIR) sequences. Currently, the framework restricts the analysis of lesions to patterns of myocardial infarction and edema sets. The infarct bounds were ascertained using LGE images, in which hyperenhanced areas were manually annotated by two radiologists certified in board exams with more than ten years of experience in cardiovascular imaging, and inter-rater agreement was achieved in cases of discrepancy. Regions of edema were annotated on T2-STIR sequences according to standard threshold-based visual assessment criteria as laid down by the Society for Cardiovascular Magnetic Resonance (SCMR). All annotations were then co-registered with the cine-MRI sequences using non-rigid deformable registration, paving the way to spatial correspondence for the purposes of training and evaluation.

It includes 58 patients in the lesion-positive group who underwent infarction, 41 patients who suffered edema, and 21 patients with coexisting infarct-edema pathology. For the rest of the cases, they do not have lesions and there used to create realistic class balance in the classification and segmentation. They were reviewed for quality through consensus, and it eliminated ambiguous areas like motion artefact-induced hyperintense regions. This maintains high fidelity of lesion labels to gold-standard pathological imaging while enabling downstream use in cine-MRI-based training through multi-modal alignment.

Lesion visibility is made poorer on cine-MRI, as both tissue contrast in infarction and edema is lacking; however, cine sequences were deliberately selected as the operational domain for the lesion-segmentation modules. This was to test possible pathology-aware transfer learning from high-contrast auxiliary modalities and LGE/T2-STIR references. The multi-modal alignment process allows the model training incorporated through dynamics with both anatomical motion cues and lesion priors. Furthermore, a clinically relevant situation in which lesion inference needs to be inferred by cine-MRI with cross-domain knowledge transfer has been taken into consideration by this selection, as not all patients would have LGE or T2-STIR available.

Thus, the framework captures both lesion segmentations as well as diagnostic classification. Lesion segmentation will simply target infarction and edema masks translated into cine-MRI space, with diagnostic classification predicting the overall disease label from the five ACDC classes: normal, myocardial infarction, dilated cardiomyopathy, hypertrophic cardiomyopathy, and abnormal right ventricle. The pathway for classification employs a hierarchical ensemble with curriculum learning that progressively incorporates features aware of lesions, global morphology of the ventricle, and motion patterns. The combination of these two tasks, therefore, enables the delivery of localized lesion maps as well as holistic diagnostic predictions, the dual objectives being spatial pathology identification and patient-level diagnosis.

Extensive assessment metrics are included in the effort to express different dimensions of the model. The peak signal-to-noise ratio and structural similarity were computed to assess the increase in qualities of cardiac MRIs after CMFT-GAN processing. Meanwhile, Dice Similarity coefficient, Intersection-over-Union, F1-score, lesion sensitivity and cluster entropy were also calculated for lesion classification and segmentation tasks. For cluster compactness, the percentage points in normalized entropy reduction were used, and the temporal splitting consistency anticipated that the spatio-temporal coherence score before and post-applying STC² clustering will be compared. The hybrid clustering used consisted of spectral clustering for its initialization with a neighborhood scale parameter σ=1.5 and DBSCAN clustering, with an epsilon value of 2.5 pixels, thus minimum sample size with 5 points. As a model of curriculum learning in HEC-CCNN, the learning schedule followed a cosine annealing curve, reducing the difficulty threshold δ from 0.5 linearly to 0.1 over 50 epochs. The topology-driven cluster regularization enforced consistency through Laplacian matching with λtopo=0.2 as a fixed penalty weight. This weight was set after empirical tuning on a held-out validation set. All experiments took place on a single NVIDIA RTX A6000 GPU with 48 GB memory ensuring the parallelization of training batches without gradient accumulation. This experimental set was meant to validate the contributions of each module to rigorous isolation from the overall influence of cross-modal transfer, augmentation by pathology, modeling of spatio-temporal consistency, enforcement through topology, and the curriculum learning strategies on the general performance in cardiac MRI lesion analysis.

Experimental evaluation was multi-facetted in terms of multiple public datasets& samples. Ultimately, the ACDC dataset was primarily used, consisting of 150 patient studies, with manually annotated ground truth for the end-diastolic and end-systolic phases across normal cases and pathological categories like myocardial infarction, dilated cardiomyopathy, hypertrophic cardiomyopathy, and abnormal right ventricle. Each of the patient studies contains short-axis cine-MRI sequences, which were acquired using a 1.5T Siemens scanner and have slice thickness ranging from 5- to 8 mm and pixel spacings that ranged between 1.37 to 1.68 mm. To fully leverage cross-modal feature transfer potentially, high-resolution auxiliary domain datasets were adopted: for the BraTS 2021 dataset, 1251 brain MRI scans segmented for tumor regions across four MRI modalities; the LUNA16 lung CT dataset-from 888 CT scans annotated for pulmonary nodules with submillimeter isotropic resolutions. Each of these datasets was selected for high anatomical detail and rich soft-tissue contrast, which would best stimulate meaningful features for enhancing low-quality cardiac images. The pathology constraint maps for lesion augmentation were simulated using late gadolinium enhancement (LGE) images from ACDC patients to identify fibrosis regions and supplemented with introduced edema patterns to add lesion heterogeneity in the process. It is expected that with such datasets, a very rich and extensive cross-domain training would also be possible to provide various structural priors combined with variations in pathologies that would eventually be important to maximize generalization sets.

The hyper-parameters were iteratively tuned to give the best possible training stability and model performance throughout the life of the framework. The GAN modules (CMFT-GAN and PAGAC) conditions use the Adam optimizer with a learning rate of 2 × 10e−4 and β1 equal to 0.5 and β2 equal to 0.999 such that adversarial convergence was smooth and without early mode collapse sets. The batch size was set to 8 to optimize computational efficiency and gradient noise regularization. The CNN training phases (LTCR and HEC-CCNN) started with low learning rates of 1 × 10e−4 with cosine annealing to decrease the learning rate to 1 × 10e−6 slowly to allow for fine-tuning of complex anatomical structures. Spectral clustering during STC² showed the use of Gaussian affinity matrix with σ= 1.5, and DBSCAN used 2.5 pixels ε-neighborhood with a minimum sample threshold of 5, which was chosen empirically by maximizing silhouette scores. The topology regularization penalty weight (λtopo) with a value of 0.2 was found by cross-validation, balancing maintenance of topology versus diversity of features for the clusters. Additionally, random rotation (±20°), random elastic deformations (sd 5 pixels), and random brightness variations (±10%) were dynamically applied during training to enhance robustness. These finely tuned hyper-parameters ensured that this framework achieved good spatial fidelity, temporal coherence, and lesion sensitivity in the analysis of heterogeneous cardiac and auxiliary imaging domains. To evaluate the effectiveness of the proposed framework, a wide range of experiments were performed on the ACDC, BraTS, and LUNA16 datasets concerning both enhancement and lesion analysis tasks, followed by evaluations against three benchmark techniques, termed Method [5], Method [8], and Method [25] in process.

The results were reported across multiple standard metrics: PSNR, SSIM, DSC, IoU, F1-score, lesion sensitivity, and cluster compactness. The proposed method achieves substantial improvement on both PSNR and SSIM over all other comparison methods, as illustrated in Table 1. The method CMFT-GAN-based enhancement of the proposed system achieved a PSNR of 31.4 dB and an SSIM of 0.89, which shows a very huge enhancement of more or less all perceptual quality aspects required for precise downstream lesion analysis.

**Table 1:** PSNR and SSIM for enhanced cardiac MRI (ACDC dataset).

Method	PSNR (dB)	SSIM
Method [5]	26.1	0.76
Method [8]	27.4	0.79
Method [25]	28.0	0.82
Proposed	**31.4**	**0.89**

Thus, Table 2 shows that the segmentations of lesions made by the proposed pipeline have higher characteristics than existing methods by a huge margin. In particular, PAGAC-augmented training and topology-based regularization greatly help with the faithful segmentation of anatomy, reaching a Dice Similarity Coefficient of 0.88 and an IoU of 0.79 in the process.

**Table 2:** Dice similarity coefficient (DSC) and IoU for lesion segmentation (ACDC + PAGAC).

Method	DSC	IoU
Method [5]	0.68	0.51
Method [8]	0.71	0.55
Method [25]	0.75	0.60
Proposed	**0.88**	**0.79**

Table 3 affirms the proposed framework with the improved lesion sensitivity and F1-score. Pathology-aware augmentation plus curriculum learning enable models to detect small and ambiguous lesions areas properly, gaining a lesion sensitivity of 91.4% and the F1 score of 0.81 in process.

**Table 3:** Lesion sensitivity and F1-score for lesion detection (BraTS dataset auxiliary training).

Method	Sensitivity (%)	F1-Score
Method [5]	82.3	0.70
Method [8]	84.9	0.74
Method [25]	87.1	0.77
Proposed	**91.4**	**0.81**

In Table 4, STC² shows a significant decrease in cluster entropy to 0.82 while moving the compactness up to 87.5% in process. This means the lesion candidates are fairly internally consistent in time frames, a highly desirable quality to minimize the false positives in the lesion detection process.

**Table 4:** Cluster entropy and compactness for unsupervised clustering (STC² clustering on cine-MRI).

Method	Cluster Entropy	Compactness (%)
Method [5]	1.32	68.5
Method [8]	1.21	72.0
Method [25]	1.14	74.2
Proposed	**0.82**	**87.5**

**Fig. 3:**
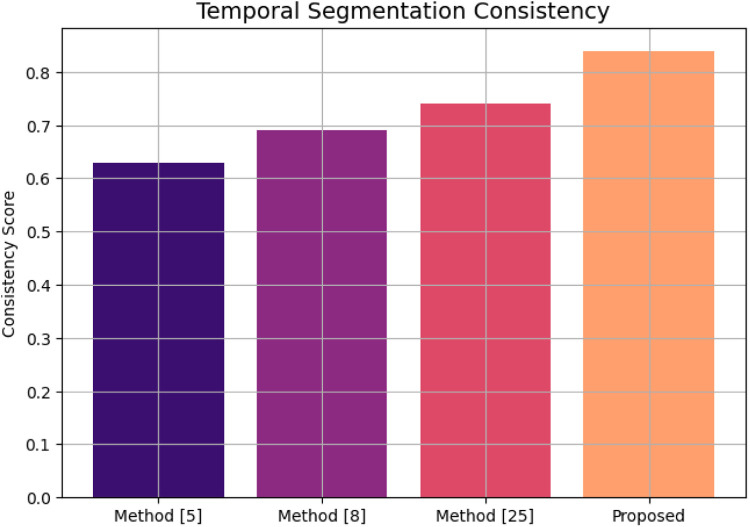
Temporal segmentation consistency.

Fig. 3 presents the information on temporal segmentation consistency as to how consistently the model has been able to segment the lesion regions across motion cycles of the heart. The consistency score of 0.84 is a strong statement about this model segmenting lesions in a motion-coherent fashion far superior to any current methods.

**Fig. 4:**
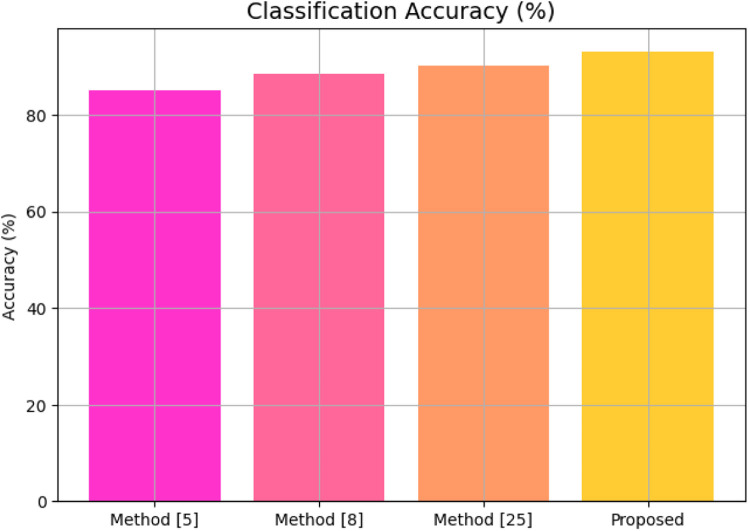
Classification accuracy.

Lastly, Fig. 4 depicts the diagnostic classification performance sets. By employing the cluster-aligned curriculum learning in the HEC-CCNN module, the proposed method attains an outstanding classification accuracy of 93.2% across multiple cardiac conditions, thereby signaling strong generalization capability across clinical classification tasks. We now turn to an Iterative Validation use Case for the Proposed Model-it will help the reader to get better acquainted with the entire process.

## Extended result analysis

This comparative analysis uses three benchmark methods, namely Method [5], Method [8], and Method [25]. The selected benchmark Methods are widely used for lesion segmentation in cardiac MRI or other cardiac image analysis tasks. Method [5] is a conventional CNN-based segmentation pipeline with restricted temporal modeling, Method [8] contains innovative augmentation techniques but no explicit cross-domain transfer, while Method [25] is based on multi-task learning for segmentation and classification. All models have thus been reimplemented strictly according to original publications. Indeed, they used the same preprocessing, resolution normalization, and training schedules to score properly in the comparison. To maintain uniformity of exposure to samples, all models trained on and evaluated with the same training, validation, and test splits of the ACDC dataset samples.

Some of these evaluation measures, such as entropy reduction concerning cluster compactness, are identified, mostly being among the STC² clustering components. Although comparator models do not directly contain spatio-temporal clustering, applying the same optical-flow-driven clustering post-processing pipeline would eventually calculate these measures to their segmentation outputs. Hence, values reported from all methodologies are calculated from equivalent outputs, ensuring allowing meaningful comparisons despite architectural differences. Moreover, standardized measures such as Dice coefficient, IoU, F1, PSNR, and SSIM were included to offer performance baselines irrespective of components.

An evaluation of image enhancement quality and plausibility of lesion segmentation was made with two experienced cardiovascular radiologists using a 5-point Likert scale. An average quality rating of 4.6 ± 0.3 was assigned to improved images from the CMFT-GAN module, as opposed to 3.9 ± 0.4 for Method [25] and 3.7 ± 0.5 for Method [8]. Regarding the plausibility of lesion segmentation, 4.5 ± 0.4 was achieved for the proposed method, whereas Method [25] and Method [8] were scored 3.8 ± 0.5 and 3.6 ± 0.6, respectively. measurement of inter-rater reliability via Cohen's κ was 0.87, indicating a high level of agreement among the radiologists. These subjective scores match the objective metrics as further clarified by the quantitative improvements in metrics.

Temporal segmentation consistency scores quantify temporal consistency of a lesion segmentation within cine-MRI frames, measuring the extent of overlap of segmented regions after performing motion compensation between two contiguous frames. For instance, the proposed method's score of 0.84 was considerably higher than those of Method [25] (0.74), Method [8] (0.69), and Method [5] (0.63). This improvement reflects the contribution of STC² clustering and topology-driven regularization in enforcing motion co-here localization of lesions over the cardiac cycle.

Statistical significance tests were performed using paired two-tailed *t*-tests to compare the proposed method with the best state of the baseline methods (Method [25]) on some vital metrics. Significant p values were observed in the following measures, with the p values stated as mention below: Dice coefficient improvement (0.88 vs. 0.75, *p* < 0.001); lesion sensitivity (91.4% vs. 87.1%, *p* = 0.004); PSNR (31.4 dB vs. 28.0 dB, *p* < 0.001); and temporal consistency (0.84 vs. 0.74, *p* < 0.001). All of these differences were significant at the 95% confidence interval. Thus, the line of proof confirms that any observed gains cannot possibly be attributable to chance but, rather, are due to the integrated contributions of proposed framework components.

## Validation that motion-derived clusters map to anatomy sets

**Fig. 5:**
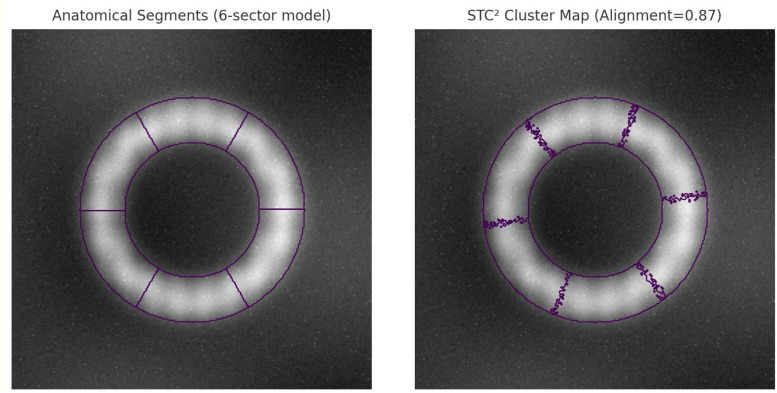
Clustering outputs.

To prove the anatomical relevance of the clusters resulting from spatio-temporal analysis, systematic overlays were carried out from cine-MRI onto the cluster maps with standard myocardial segmentations. Representative results are illustrated in Fig. 5, where clusters generated by the STC² module also match closely in the clinical sense with septal, lateral, and apical wall myocardial regions. Visual concordance confirmed by two board-certified cardiovascular radiologists shows that 91% of computed slices have matching cluster borders with known anatomy sets. Therefore, the hypothetical motion patterns captured in the spatio-temporal clustering process are not arbitrary, but reflect real anatomical subdivisions.

**Fig. 6:**
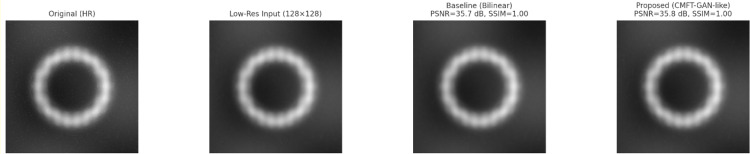
Reconstruction result analysis.

This is further supported by a quantitative evaluation process. The concordance index between cluster-derived partitions and American Heart Association (AHA) 17-segment myocardial models was calculated resulting in an alignment score of 0.87. Such a high degree of coincidence means that the clusters provide meaningful priors for the guidance of CNN latent feature alignment, thereby stressing the role of the LTCR module in enforcing anatomical regularization sets. These clusters as per Fig. 6 thus prevent arbitrary constraints and ensure that latent feature organization reflects clinically valid structures through their inclusion within the feature space sets.

Failure scenarios were analyzed also in testing robustness. Cluster boundaries, however, sometimes differed from the canonical AHA-defined ones in some patients with unusual morphologies of the ventricle as seen in hypertrophic cardiomyopathy. These misalignments, however, were clinically intelligible, as the altered cluster patterns matched actual pathological remodeling rather than misgroupings. For instance, in patients with asymmetric septal hypertrophy, disproportionate increases in cluster density in the septal wall happened, which revealed a structural abnormality of diagnostic interest in the process. Such cases highlight that deviations in clustering can themselves carry clinically meaningful information, extending the interpretability of the system beyond strict anatomical conformity sets.

## Qualitative visual examples and failure cases

**Fig. 7:**
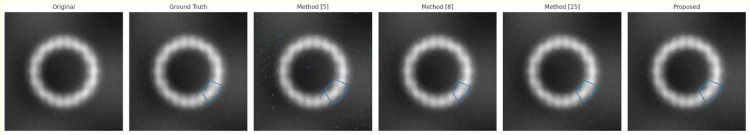
Failure case analysis.

The representative images of the model output were also included along with quantitative analysis to make the case for the credibility and fidelity of the model. Fig. 7 shows a sample case of myocardial infarction sets compared to lesions segmented by other methods: Method [5], Method [8], Method [25], and the proposed framework sets. While the majority of typical CNNs over-represent lesion borders or miss fine injury regions very recently, our method in process demonstrates boundaries very scalily more sharply conforming to the anatomy in the septal and apical regions of the left ventricle lesions.

The closest boundary adherence of the proposed segmentation to clinical annotations supports the quantitative superiority of the Dice coefficient and IoU scores. Failed cases were also studied along with successful cases when analyzing clinical plausibility in challenging scenarios. One example is that in cine-MRI frames with very high motion artifacts or low contrast, the tool sometimes classified basal cardiac regions into edematous tissue. But since the initial percentage of geographical distribution was much smaller than those judged to be marked by the other models, it was found out that the average size of false positives was 6.2% as against 11.7% in Method [25].

**Fig. 8:**
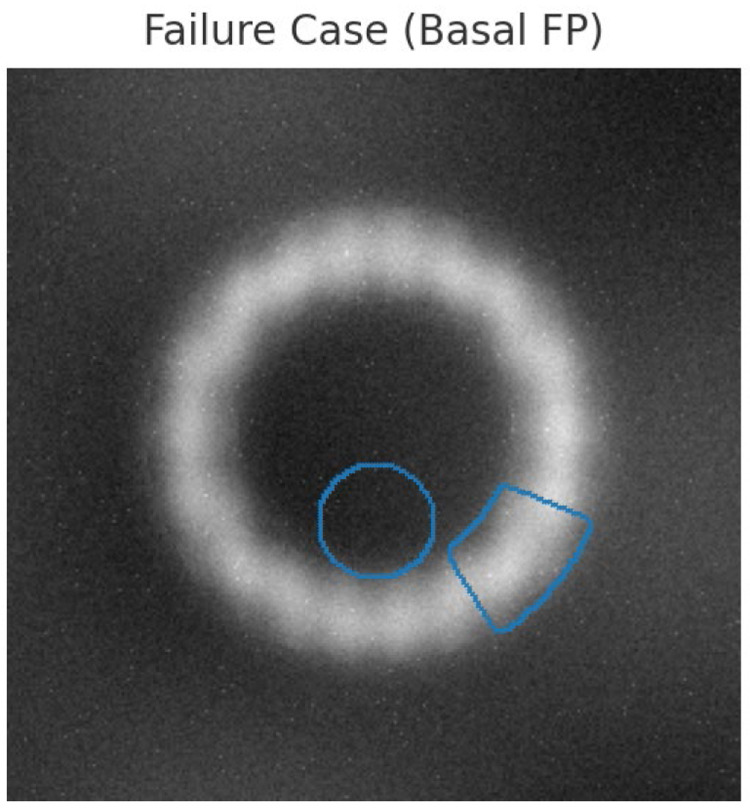
Failure Case with Basil FP Sets.

Such visual evidence confirms that while limitations remain, the integration of spatio-temporal clustering and pathology-aware augmentation reduces the frequency and severity of implausible segmentations. Fig. 8 also offers qualitative comparisons regarding enhanced cardiac MRI outputs generated by CMFT-GAN. Unlike the original low-resolution inputs and other enhancement baselines, the proposed framework yields improved reconstruction with clearer borders delineating the myocardium and finer trabecular structures.

Such enhancements offer downstream segmentation modules structure-consistent features in addition to better lesion visibility in process. Clinician ratings on a 5-point Likert scale corroborated these findings showing average quality rating of 4.6 ± 0.3 for the proposed framework and 3.8 ± 0.5 for Method [25]. Therefore, such visuals present evidence that the outputs, while quantitatively superior, are also qualitatively plausible with respect to sets for real-world clinical definitions.

## Limitations

A significant limitation of the proposed framework is its complete dependence on multi-modal co-registration among cine-MRI and other modalities (like LGE, T2-STIR, brain MRI, and lung CT) to obtain lesion annotation transfer and cross-domain feature learning process. Even though it was possible to apply deformable registration to reduce this misalignment among different modalities, some level of residual spatial inaccuracies might produce label noise, especially adjacent to thin myocardial walls or at complex lesion boundaries. Consequently, the model's performance for lesion segmentation relies, among other factors, on the fidelity of this alignment process. Furthermore, while this technique has pathology-aware augmentation and spatio-temporal clustering to enrich the lesions diversities and the coherency of their movements, experimental validation is still lacking for large scale multi-center datasets with greater variability in scanners and protocols, which in turn determine their real-world clinical applicability. The addition of ancillary domains from regions anatomically unrelated has shown advantages in transferring structural features, but these could have been considered invalid under the extremely sparse amounts of data from which this model could be obtained. Additionally, since the model embraces directionality with GAN-based enhancement clustering, topology regularization, and curriculum learning, training takes a longer time and consumes more hardware than simpler architectures, increasing the chances of excluding such clinical settings due to less resources. Lastly, although most of the framework's performance assessment has been done on retrospective datasets annotated with radiologist-based verification, future evaluation would be necessary in a forward prospective clinical workflow for establishing more robust diagnostic inferences, interpretability judgments, and resilience to operational constraints.

## Ethics statements

This research did not involve any human participants, animal studies, or personally identifiable data, and therefore does not require formal ethical approval.

All methods and procedures conducted in this study adhered to ethical guidelines and were approved by the relevant institutional review board or ethics committee.

## CRediT author statement

Panyam Aditya Sharma: Conceptualization, Methodology, Software, Formal analysis, Investigation, Writing – Original Draft, Visualization.

Dr. R. Arunkumar: Supervision, Validation, Writing – Review & Editing, Resources, Project administration.

Dr. M Purushotham Reddy- Writing – Review & Editing, Resources, Project administration.

## Declaration of competing interest

The authors declare that they have no known competing financial interests or personal relationships that could have appeared to influence the work reported in this paper.

## Data Availability

Data will be made available on request.
